# Liposome Entrapment of Bacteriophages Improves Wound Healing in a Diabetic Mouse MRSA Infection

**DOI:** 10.3389/fmicb.2018.00561

**Published:** 2018-03-29

**Authors:** Sanjay Chhibber, Jasjeet Kaur, Sandeep Kaur

**Affiliations:** Department of Microbiology, Panjab University, Chandigarh, India

**Keywords:** bacteriophage, diabetes, phage cocktail, liposome, alloxan

## Abstract

Diabetic populations are more prone to developing wound infections which results in poor and delayed wound healing. Infection with drug resistant organisms further worsen the situation, driving searches for alternative treatment approaches such as phage therapy. Major drawback of phage therapy, however, is low phage persistence *in situ*, suggesting further refinement of the approach. In the present work we address this issue by employing liposomes as delivery vehicles. A liposome entrapped phage cocktail was evaluated for its ability to resolve a *Staphylococcus aureus*-induced diabetic excission wound infection. Two characterized *S. aureus* specific lytic phages, MR-5 and MR-10 alone, in combination (cocktail), or entrapped in liposomes (versus as free phages) were assesed for their therapeutic efficacy in resolving diabetic wound infection. Mice treated with free phage cocktail showed significant reduction in wound bioburden, greater wound contraction and faster tissue healing than with free monophage therapy. However, to further enhance the availability of viable phages the encapsulation of phage cocktail in the liposomes was done. Results of *in vitro* stability studies and *in vivo* phage titer determination, suggests that liposomal entrapment of phage cocktail can lead to better phage persistence at the wound site. A 2 log increase in phage titre, however, was observed at the wound site with liposome entrapped as compared to the free phage cocktail, and this was associaed with increased rates of infection resolution and wound healing. Entrapment of phage cocktails within liposomes thus could represent an attractive approach for treatment of bacterial infections, not responding to antibiotis as increased phage persistence *in vitro* and *in vivo* at the wound site was observed.

## Introduction

Diabetes is a chronic disease that manifests in the form of a chronic hyperglycemia which is associated with a plethora of complications including visual impairment, blindness, kidney disease, nerve damage, amputation, heart disease, and stroke (Loghmani, 2005). WHO has projected diabetes as the 7th leading contributor to death by 2030 (Mathera and Loncar, 2006). In addition, the total number of people with diabetes is projected to rise from 171 million in 2000 to 366 million in 2030 (Wild et al., 2004). Diabetic patients are five times more susceptible to fungal and bacterial infections (Axelrod, 1985). This is due to several factors that include a high glucose level which encourages bacterial growth. Secondly, diabetes decreases a body’s blood flow, slowing healing of abrasions, open wounds and other injuries, thus making diabetic individuals more prone to infection (King, 2001). Finally, peripheral neuropathy, a primary complication of diabetes, results in loss of sensation (Pendsey, 2010) and has a central role in the development of foot and wound infections (Bader, 2008; Powlson and Coll, 2010). According to the American Diabetes Association (ADA), 25% of people with diabetes suffer from a wound problem during their lifetime, including infections. *Staphylococcus aureus* is one of the most common pathogens isolated from wounds in diabetic patients. Further, with the spread of methicillin resistant strains of *S. aureus* (MRSA), management of such infections in diabetic population has become challenging. Diabetics who contract MRSA are at a greater risk of harboring more serious, slow-healing wounds (Rogers and Bevilacqua, 2009).

With time, the bacteria in diabetic infections tend to evolve greater resistance to antibiotics (Xavier et al., 2014). Consequently, having a comprehensive treatment plan for MRSA is crucial. Phage therapy seems to be a good option for addressing these problems (Barrow and Soothill, 1997; Bull et al., 2002; Wills et al., 2005; Capparelli et al., 2007; Sunagar et al., 2010; Hsieh et al., 2011; Kumari et al., 2011; Chhibber et al., 2013; Chadha et al., 2016). Fish et al. (2016), on the basis of the success obtained with phage therapy of a diabetic foot infected with *S. aureus*, have suggested that in the future controlled clinical trials can be conducted. The ability of phages to rapidly lyse infected bacteria, their specificity and effectiveness against multidrug-resistant pathogenic bacteria, their ability to self-replicate (auto dosing) (Kumari et al., 2009; Chhibber et al., 2013; Kaur et al., 2014), their natural abundance and their proven clinical safety makes this therapy worth considering in immunocompromised individuals such as diabetic patients (Carlton et al., 2005; Hanlon, 2007). Phage therapy in many cases nevertheless can benefit from approaches that can increase phage retention and persistence at the target sites.

Toward improving phage persistence and retention at diabetic wound sites, we propose here the use of liposome entrapment of phages. Liposomes are composed of natural lipids which are biodegradable, non-immunogenic and non-toxic. Along with these advantages, their structural versatility enables us to design a number of liposome-based formulations (Gregoriadis, 1995). Liposomes also mimic biological membranes in terms of their structure and behavior, which enables them to penetrate the epidermal barrier to a greater extent and thus can be used effectively in treating skin infections (Hua, 2015). Use of drug delivery systems (DDSs) such as liposomes also helps to improve the pharmacological properties of conventional (“free”) drugs by altering their pharmacokinetics (PK) and bio-distribution (BD) (Zhang et al., 2013; Zylberberg and Matosevic, 2016). Workers in the past have reported the use of liposomes with entrapped antibiotics or other wound healing agent in treating wound infections (Roesken et al., 2000; Kurilko et al., 2009; Fukui et al., 2012). In recent studies, entrapment of phages in liposomes has also been reported (Colom et al., 2015; Nieth et al., 2015; Singla et al., 2015; Chadha et al., 2017). However, none of these workers have studied the use of liposomes to enhance the stability and persistence of phages for the better therapeutic outcome after treatment of diabetic wound infection caused by MRSA. The present study therefore investigated the therapeutic use of liposome entrapped phage cocktail in treating MRSA mediated skin wound infection in diabetic mice.

## Materials and Methods

### Bacterial Strains and Phage Used

*Staphylococcus aureus* ATCC 43300 (MRSA) from ATCC, Manassas, VA, United States was used in this study. *S. aureus* specific phages, MR-5 and MR-10, both of which have been isolated and characterized in our laboratory as lytic, dsDNA, tailed phages belonging to Myoviridae family, order Caudovirales were used in the present study (Kaur et al., 2012; Chhibber et al., 2013).

### Preparation of Purified High Titer Phage Stocks (MR-5 and MR-10)

High-titer MR-5 and MR-10 suspension was prepared according to the method described by Langley et al. (2003). The inoculated broth was centrifuged and supernatant containing phage collected and passed through a 0.22-μm-pore-size filter. The filtrate was concentrated by using Millipore Labscale TFF system (Pellicon) and 10 K (polyethersulfone) membrane until the sample volume was reduced to 25–30 ml. DNase I (0.25 mg/ml) was added and kept for 1 h at 37°C to digest the free DNA. NaCl (final concentration of 1 M) and polyethylene glycol (PEG) 8000 were added to the concentrated lysate and kept at 4°C overnight. The precipitate was collected, dialyzed against phosphate buffered saline (PBS) overnight at 4°C, passed through an0.22-μm-pore-size filter and stored at 4°C till further use. The phage titer was determined in the final product.

#### Liposomes

Free phage preparations were entrapped within liposomes to increase their persistence and efficacy. For this a series of optimization experiments were carried to select the exact liposomal formulation for *in vivo* use.

##### Preparation

The method of Bhatia et al. (2004) was used for the preparation of phage cocktail-loaded liposomes. For preparation of cationic liposomal formulation of phage, phosphatidylcholine:cholesterol: tween 80:stearylamine (7:3:1:0.5) was dissolved in chloroform-methanol mixture (2:1 v/v). Thin film was prepared by a rota-evaporator [Heidolph, Hei-VAP Advantage (ML)] using a hydration temperature of 40°C. Phage suspension (10 ml prepared in PBS [pH 7.2]) was added to the thin film at 40°C and rotated for 10 min to detach the film from the glass wall. The suspension was left overnight at room temperature for swelling. The next day the dispersion is sonicated in a water bath sonicator for 30 min.

##### Characterization

The morphological characteristics of liposomes were monitored viz. shape, uniformity, and structure by using optical microscope (Olympus CH20i) at suitable magnification (100X). Morphological analysis of liposomes loaded with phage cocktail was then done using TEM following the procedure of Goodridge et al. (2003). Drops of ultra-centrifuged phage samples (1,00,000 *g* for 2 h, 4°C; L-80, Beckman Instrument, Switzerland) were dropped on nitrocellulose coated grids (diameter, 3 mm; 300 meshes), stained with 2% (w/v) potassium phosphotungstate (pH: 6.8–7.2) for 10 s, and examined under a transmission electron microscope (TEM) (Hitachi H 7500, Tokyo, Japan) at Sophisticated Analytic Instrumentation Facility (SAIF), Panjab University, Chandigarh, India. Particle size and polydispersity index of liposomes was measured by dynamic light scattering (DLS) technique on Beckman coulter instrument (Delsa Nano C) at Department of Chemistry, Panjab University, Chandigarh.

The phage entrapment efficiency of liposomes was determined by modified protamine aggregation method (Gulati et al., 1998). Total phage count was determined by adding Triton × (0.02%) in a ratio of 1:1 v/v so as to disrupt liposomes and release entrapped phage particles. The mixture was then centrifuged (10,000 ×*g* for 10 min) and total number of phages in the supernatant was determined using plaque assay. Free phages were separated by protamine aggregation method where protamine sulfate was added at a final concentration of 20 mg/ml. The liposomal phage preparation with added protamine sulfate was allowed to stand overnight. Supernatant was collected and the number of free phage particles was determined using plaque assay. Phage entrapment efficiency was then calculated as total number of non-free phages divided by total number of phages, presented as a percentage.

##### Animal experiments

Animal experimental protocols were approved by the Institutional Animal Ethics Committee (Approval ID: IAEC/411) of Panjab University, Chandigarh, India and performed in accordance with the guidelines of Committee for the Purpose of Control and Supervision of Experiments on Animals (CPCSEA), Government of India, on animal experimentation. All efforts were made to minimize the suffering of animals.

BALB/c female mice, 4–6 weeks old weighing 20–25 g were used in this study. The animals, obtained from Central Animal House, Panjab University, Chandigarh, India, were kept in polycarbonate cages housed in well-aerated rooms with a 12-h light/12-h dark cycle at 25 ± 2°C, and fed with standard rodent diet and water *ad libitum*. Diabetes as per the protocol of Pan and Liu (2008) was induced by giving two intra-peritoneal injections of alloxan monohydrate (150 mg/kg body weight) at 48-h intervals in mice fasted overnight. One week after the second injection, blood glucose levels (both random as well as fasting) were recorded. The blood sample was obtained by tail clipping method of Oladeinde et al. (2007) and glucose levels were checked using pre-calibrated Glucometer (Optium Xceed).

#### Wound Model

A diabetic excision wound model was developed following the method of Mendes et al. (2012). *S. aureus* 43300 cultivated overnight in brain heart infusion broth was pelleted and washed twice with PBS. Bacterial suspension was adjusted to achieve a cell density corresponding to a range of bacterial inoculums (10^8^, 10^9^, and 10^10^ CFU/ml). The number of CFU/ml was confirmed by quantitative plate count. Diabetic BALB/c mice were taken and distributed in four different groups of 14 animals (*n* = 14) each. The mice were anesthetized by giving 0.1 ml of ketamine-xylazine (50–5 mpk) intraperitoneally. Hair was removed by using commercially available hair removing cream followed by disinfection of the dorsal surface with 70% alcohol. A punch biopsy instrument (diameter 5 mm, accu punch) was used to create a full thickness round wound extending through the panniculus carnosus. The wounds created in all the animals were left open and animals were given ibuprofen suspension (20 mg/ml) in drinking water. Each of the three groups received different doses of inoculum. Animals of the fourth group received same volume of PBS injected into their wound. Two mice from each group were sacrificed on days 1, 3, 5, 7, 10, 15, and 20 post-bacterial challenge by cervical dislocation. Complete epithelial and dermal compartments of wound margins, granulation tissue and adjacent muscle and subcutaneous fat tissue was excised from each individual wound and processed. The tissue was homogenized and dilutions of the homogenates were plated to determine the bacterial burden.

#### Free Phage Efficacy (Study 1)

*In vivo* work was divided into two studies. In the first study the therapeutic potential of free phages (monophage therapy as well as cocktail of phages) not involving any delivery system was assessed. For further clarity, phage preparations not entrapped within liposomes have been referred as free monophage (FMP) for, e.g., FMP-MR-5 and FMP-MR-10 and free cocktail of phages (FCP).

For the first study, the therapeutic potential of *S. aureus* 43300-specific phages FMP-MR-5, FMP-MR-10 and their cocktail, FCP, was evaluated in diabetic BALB/c mice. The phages were administered locally at a dose of 10^9^ PFU/50 μl, at MOI10 as based on a titer of applied phages of 2 × 10^10^ PFU/ml, into the wound on its dorsal surface. Diabetic BALB/c mice were randomly divided into five groups (*n* = 14 per group; as described immediately below) and seven extra animals were put in the infection control group to check the bacterial load on days 1 and 3. In all cases diabetic mice were first infected with *S. aureus* 43300 (10^8^ CFU/50 μl) with treatments following 30-min later.

Group 1 (untreated infection control): No phages or clarithromycin applied.
Group 2: Local administration of phage FMP-MR-5 at MOI10.
Group 3: Local administration of phage FMP-MR-10 at MOI10.
Group 4: Local administration of the phage cocktail, FCP (each phage at MOI10 mixed in an equal ratio).
Group 5: Two I/P doses of clarithromycin (10 mpk) given in 12-h intervals.

All of the animals involved in Study 1 were monitored daily for mortality post-bacterial challenge. In addition to mortality, wound bioburden, and phage titer was assessed in surviving animals on different days.

#### Liposome Entrapped Phage Efficacy (Study 2)

In the second study, liposome entrapped phage cocktail was evaluated for its potential in treatment of diabetic wound infection. Phage cocktail (MR-5 + MR-10, 1:1) entrapped within liposomes shall be referred as liposomes entrapped cocktail of phages (LCP). Results were compared with the groups instead treated with FCP and with clarithromycin. Diabetic BALB/c mice with dermal wounds in this study were randomly divided into a total of four groups (*n* = 14). As above, in all cases diabetic mice were first infected with *S. aureus* 43300 (10^8^ CFU/50 μl) with treatments following 30-min later.

Group 1 (untreated infection control): No phages or clarithromycin applied.
Group 2: Local administration of FCP (each phage at MOI10 mixed in an equal ratio).
Group 3: Local administration of LCP (each phage at MOI10 mixed in an equal ratio).
Group 4: Two I/P doses of clarithromycin (10 mpk) given in 12-h intervals.

All animals in Study 2 were monitored daily for mortality post-bacterial challenge. In addition to mortality, wound bioburden, phage titer, wound contraction rate, MPO, Masson’s trichrome staining, histopathological examination, and period of epithelization was also assessed in surviving animals on different days.

##### Wound microbiology and rate of healing

In both Studies 1 and 2, two mice from each group were sacrificed on days 1, 3, 5, 7, 10, and 15 post-infection by cervical dislocation. Bacterial load (wound bioburden) was assessed as per the method of Park et al. (2009). After disinfection, the wound tissue was carefully excised and homogenized in 1 ml of PBS. This tissue homogenate was then serially diluted and plated for CFUs. Phage titer was determined (in terms of PFU/ml) by centrifuging the homogenate (10,000 *g* for 10 min at 4°C). The supernatant was filtered through 0.45 μm pore sized filter and the filtrate so obtained was serially diluted and plated on a lawn of *S. aureus* 43300.

Rate of wound contraction, as a percentage, was calculated by measuring the size of the wound (diameter in mm) on each day of all the animals involved in Study 2 using vernier caliper. This data is presented as percentage decrease in wound size. Thus, (initial wound size – wound size on a specific day) × 100. The number of days required for wound healing and eschar to fall off leaving no raw wound behind was taken as period for epithelization.

##### Myeloperoxidase (MPO) estimation

Two mice from each group (same groups as those described for liposome entrapped phage cocktail protection study, i.e., Study 2 with 14 animals per group) were sacrificed and their wound tissue homogenized. Homogenized samples were processed for MPO determination as per the method of Greenberger et al. (1996). The absorbance was read immediately at 490 nm over a period of 4 min. MPO was calculated as the change in optical density (O.D) × dilution factor (D.F).

##### Masson’s trichrome staining

To assess the wound healing after treatment with LCP, FCP and clarithromycin, collagen formation was checked in each animal group of Study 2. Collagen is used as an essential marker in wound healing studies as it restores the integrity of the skin. Therefore, collagen formation was observed in the skin wound tissue on different days. Samples were fixed on different glass slides, stained as per the protocol^1^ and examined under an Olympus microscope (at 100X) to evaluate the amount of collagen formation.

##### Histopathological examination

Tissue damage and healing following treatment with LCP, FCP, and clarithromycin in Study 2 was assessed on the basis of histopathological examination of infected wound tissue according to the method of Brans et al. (1994). The sections were picked on separate slides, stained with hematoxylin and eosin (Hi-Media, Mumbai) and then examined under a microscope to evaluate the extent of tissue damage and recovery.

### Statistical Methods

All the data is expressed as mean ± standard deviation of more than two experimental values for every variable. The statistical significance of difference between groups was determined by Student’s *t*-test (two groups), one-way ANOVA followed by a Tukey test using Sigma Stat, Graph pad prism (GraphPad Software, San Diego, CA, United States). *P*-value of less than 0.05 was considered statistically significant.

## Results

### Optimization of Liposomal Formulation and Stability

**FIGURE 1:**
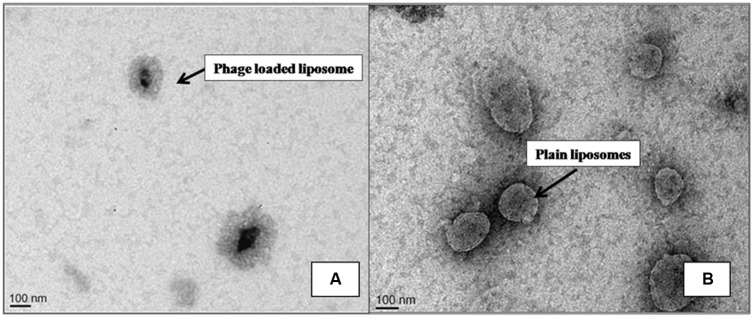
Transmission Electron Micrograph of liposomes. Photomicrograph **(A)** shows phage loaded liposomes. **(B)** Plain liposomes without phage.

For phage cocktail entrapment, three different liposomal formulations (7:3:1, 8:2:1, 9:1:1) were made using different ratios of phospholipid phosphatidylcholine (PC), cholesterol (CHOL), and Tween-80. Further, to increase the physical stability of liposomal preparation; positive charge inducer stearylamine (SA) was added in a ratio of 7:3:1:0.5. Liposomal formulation 7:3:1:0.5 showed a minimum liposome size of 230 nm, low PDI of 0.220, uniform liposomes in terms of shape and lamellarity. Both the phages MR-5 and MR-10 (1:1) were entrapped in this liposomal preparation which had an entrapment efficiency of 87% and liposomal size of 212 nm. The photomicrographs (**Figure 1**) obtained after TEM clearly demonstrates presence of phages in the cationic liposomes. LCP was made using 7:3:1:0.5 formulation and tested for stability (drop in phage titer) over a 9 week period. This liposomal preparation had a PDI below 0.3 with minimum size variation and was found to be stable at 4°C in terms of fusion size, aggregation and number of entrapped phages during the entire storage period. Thus, this ratio was selected for further *in vivo* experiments.

#### Induction of Diabetes in Mice

The average random blood glucose level of normal BALB/c mice was found to be 139 ± 3.8 mg/dl whereas the fasting blood glucose level (after overnight fasting) was 96.2 ± 7.9 mg/dl. Fasting glucose levels were checked daily post-alloxan injection. Mice with fasting blood glucose levels in the range of 150–180 mg/dl, 4–5 days post-injection were termed as moderately diabetic. However, 15 days after alloxan administration, the fasting blood glucose level was found to be 194.6 ± 17.1 mg/dl, whereas the random blood glucose levels were ≥400 mg/dl. Such animals were considered severely diabetic and selected for infection studies.

#### Excision Wound Infection Model

The excision wound model was developed by inflicting full thickness wound of 5 mm extending through panniculus carnosus with the help of punch biopsy on the dorsal surface of mouse. Since the aim of this study was to assess the efficacy of phages in reducing the mortality and clearing off infection; a high dose of 10^8^ CFU/50 μl of *S. aureus* 43300 was chosen as the optimal infectious dose. At this bacterial dose, an acute infection of the inflicted wound was established with 70% of untreated mice exhibiting mortality within 24–48 h post-infection.

#### Phage Protection Studies

##### Mortality vs. survival

Animals in all groups were monitored daily for mortality. Untreated mice of Study 1 (group 1) and Study 2 (group 1) exhibited 70% mortality within 24–48 h post-inoculation. However, all treated animals showed no mortality during the experimental period and all infections resolved. This clearly indicated that a single injection of free phages, alone or as cocktail, or liposome-entrapped phages were able to protect the test animals from death.

##### Wound microbiology

The wound bioburden (bacterial density) and phage titer was also assessed in all the mice groups of Study 1 and Study 2. In Study 1, untreated animals showed an increase in bacterial load that reached ∼9 log CFUs/ml by day 3 (peak day). Mice receiving FMP-MR-5, FMP-MR-10, i.e., groups 2 and 3 respectively, showed bacterial loads which instead were 7 log CFUs/ml on day 1 and day 3 followed by a significant decline (*P* < 0.05) to 3 logs on day 10 and sterile wounds by day 15 (**Table 1**). However, mice treated with cocktail, i.e., FCP showed higher reduction in bacterial burden as compared to infection control as well as monophage treated mice on all days. A significant decrease of 3 log (*P* < 0.05) was observed in cocktail treated group on days 3 and 5 as compared to infection control. Clarithromycin treated mice also showed significant reduction on all days with sterile wounds obtained by day 10.

**Table 1:** Bacterial load (in terms of Log CFU/ml) in skin wound of *S. aureus* 43300 (10^8^ CFU/50 μl) infected diabetic BALB/c mice following treatment with FMP (MR-5 and MR-10), FCP and clarithromycin (10 mg/kg/ per i.p.).

Treatment groups (*n* = 14, each group)	Bacterial load (Log CFU/ml) on				
	Day 1	Day 3	Day 5	Day 7	Day 10
Infection control (group 1)	8.37 ± 0.07	9.24 ± 0.26	8.44 ± 0.40	6.84 ± 0.08	NA
FMP-MR-5 (group 2)	7.67 ± 0.8	7.2 ± 0.20	6.29 ± 0.17	5.21 ± 0.38	3.2 ± 0.54
FMP-MR-10 (group 3)	7.69 ± 0.14	7.11 ± 0.20	6.23 ± 0.15	5.14 ± 0.25	3.11 ± 0.31
FCP (group 4)	7.24 ± 0.25	6.83 ± 0.28	5.2 ± 0.40	4.42 ± 0.14	2.10 ± 0.36
Clarithromycin (group 5)	7.16 ± 0.40	6.5 ± 0.36	4.82 ± 0.07	3.18 ± 0.10	(–)

*Treatment was given after 30 min of wound inoculation. (-): zero bacterial count; NA: no mice available for determining the wound bio-burden. All values represent mean ± standard error of the mean calculated from three independent values.*

**FIGURE 2:**
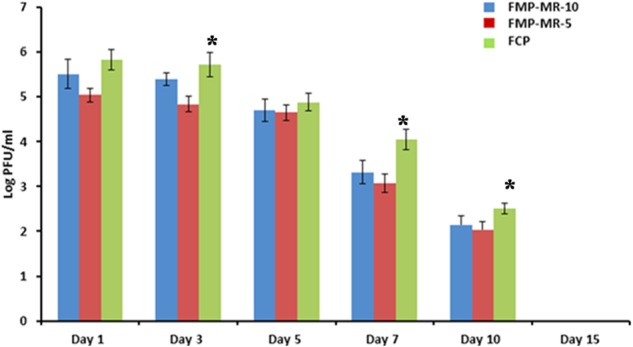
Phage titer (Log PFU/ml) in the wound of *Staphylococcus aureus* 43300 (10^8^ CFU/50 μl) infectedmice (*n* = 14, each group) following treatment with FMP-MR-10, FMP-MR-5, and FCP on different days post-infection. Error bars represent the standard deviation (SD) from three independent values. ^∗^*P* < 0.05 indicate statistical significant differences (Student’s *t*-test) between FMP and FCP treated groups.

Phage titer for Study 1 is shown in **Figure 2** was also determined on subsequent days post-treatment. Although an initial titer of 10^9^ PFU/50 μl (MOI 10) was administered at the wound site, maximum titer of 10^5^ PFU/ml was estimated on day 1, which shows a 4 log reduction from the initial phage titer. This phage count was consistent (approximately 10^5^ PFU/ml) in all the groups on days 1 and 3, followed by gradual decline on subsequent days with minimum count of 10^2^ PFU/ml present on day 10. However, phage titer was higher on all days in the wound of mice receiving FCP as compared to monophage treated animals. No phage particles were detected in the wound of mice receiving either single or cocktail of phages by day 15.

**FIGURE 3:**
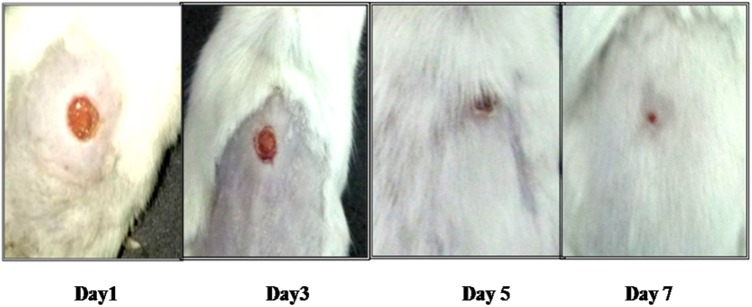
Pictorial representation of wound healing in diabetic BALB/c mice infected with 10^8^ CFU/50 μl dose of *S. aureus* 43300 and treated with LCP.

Results in **Table 2** for Study 2 show that the wounds of mice in the infection control (group 1) also had consistently higher bacterial burden (8–9 log CFU/ml) till day 5 followed by decline from day 7 onwards. In FCP treated mice, a significant decrease of 3 log CFU/ml occurred on day 3 and day 5 itself as compared to untreated mice. However, bacterial load (2.21 log CFU/ml) was detected even by day 10. In case of LCP treated mice maximum reduction in bacterial load equivalent to ∼4 log CFU/ml (*P* < 0.05) was seen on day 3 (peak day of infection). Negligible counts were obtained on day 7 and this correlated well with visible reduction in wound size by day 7 (**Figure 3**). The wound showed complete healing within 9 days unlike 20 days and less than 15 days required for untreated surviving diabetic mice and FCP treated group, respectively. Animals receiving clarithromycin showed higher reduction in bacterial burden on all days as compared to FCP treated mice and reduction in bacterial load was comparable to that seen in LCP treated animals.

**Table 2:** Bacterial load (in terms of Log CFU/ml) in skin wound of *S. aureus* 43300 (10^8^ CFU/50 μl) infected diabetic BALB/c mice following treatment with FCP, LCP, and clarithromycin (10 mg/kg/per i.p.).

Treatment groups (*n* = 14, each group)	Bacterial counts (Log CFU/ml) on				
	Day 1	Day 3	Day 5	Day 7	Day 10
Infection control (group 1)	8.39 ± 0.72	9.08 ± 0.32	8.9.32	6.16 ± 0.27	NA
FCP (group 2)	7.22 ± 0.22	6.78 ± 0.30	5.15.38	4.57 ± 0.44	2.21 ± 0.20
LCP (group 3)	6.22 ± 0.20	5.53 ± 0.41	3.28 ± 0.28	1.84 ± 0.17	(–)
Clarithromycin (group 4)	7.04 ± 0.15	6.28 ± 0.17	4.75 ± 0.45	2.92 ± 0.30	(–)

*Treatment was given after 30 min of wound inoculation. (-): zero bacterial count; NA: no mice available for determining the wound bio-burden. All values represent mean ± standard error of the mean calculated from three independent values.*

**FIGURE 4:**
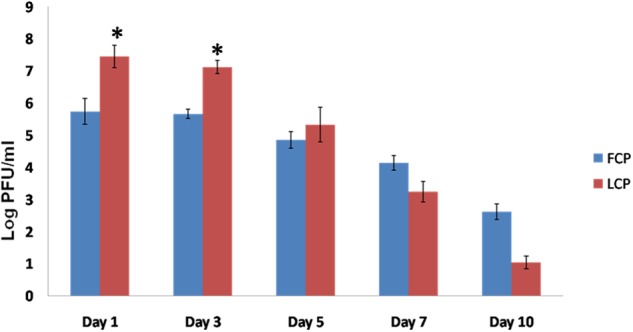
Phage titer (Log PFU/ml) present in the wound of *S. aureus* 43300 (10^8^ CFU/50 μl) infected mice (*n* = 14, each group) following treatment with FCP and LCP on different days post-infection. Error bars represent the standard deviation (SD) from three independent values. ^∗^*P* < 0.05 indicate statistical significant differences (Student’s *t*-test) between FCP and LCP treated groups.

Phage titer in Study 2 in terms of log PFU/ml (**Figure 4**) was maximum on day 1 and thereafter a decrease was noticed on subsequent days in both FCP and LCP treated groups. Maximum titer of 7.45 and 7.12 log PFU/ml on day 1 and day 3 was seen in liposome phage entrapped cocktail (LCP) treated mice as compared to 5.80 and 5.68 log PFU/ml present on day 1 and day 3 in FCP treated group. A decline was seen thereafter due to clearance of its host bacteria as no phage particles were obtained on day 15 and after day 10 with FCP and LCP treated groups, respectively. This shows that entrapment of liposomes led to a significant increase in phage titer by 2 log (*P* < 0.05) at the wound site, which explains the faster wound healing process.

#### Additional Parameters (Study 2)

##### Wound contraction

**FIGURE 5:**
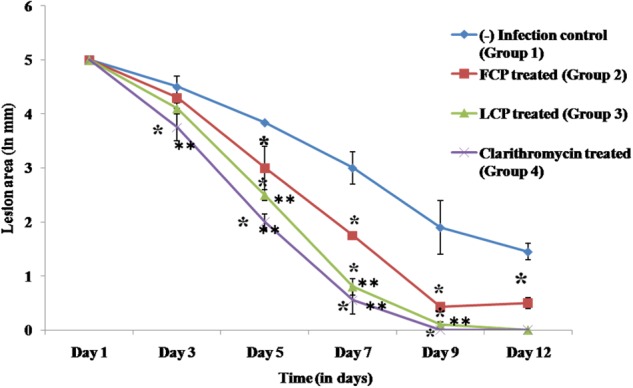
Biometric analysis (mm) of wound area of *S. aureus* 43300 (10^8^ CFU/50 μl) infected animals (*n* = 14, each group) following treatment with FCP, LCP, and clarithromycin. Lesion area was measured on various days post-infection. Error bars represent the standard deviation (SD) from three independent values. ^∗^*P* < 0.05 indicate statistical significant differences (Student’s *t*-test) between infected control and treated groups. ^∗∗^*P* < 0.05 indicate statistical significant differences between FCP treated and other treated groups (i.e., LCP and clarithromycin treated).

Wound area of all the animal groups was measured with the help of vernier caliper (**Figure 5**). In untreated mice (group 1), an initial wound size of 5 mm persisted until day 3 and it was finally resolved by day 20. In FCP treated animals wound contraction was comparatively slower with wound of 3 mm size evident by day 5 and even by day 12 a wound size of 0.5 mm was observed. However, in case of both LCP treated as well as clarithromycin treated animals, significant decrease in wound size was seen by day 3 onwards as compared to both infection control and FCP treated mice. Minimal wound size of less than 1 mm was observed by day 7 with almost complete closure of wound by day 9 in clarithromycin as well as LCP treated mice. Thus wound closure rates were greater given when phages were encapsulated than when they were not.

##### Myeloperoxidase (MPO) activity

**FIGURE 6:**
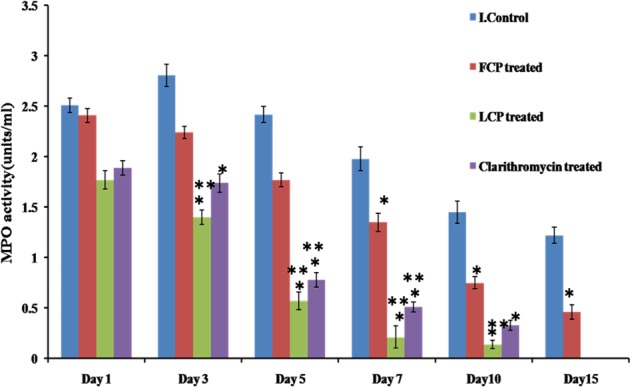
Comparison of MPO levels in the wound tissue of *S. aureus* 43300 (10^8^ CFU/50 μl) infected animals (*n* = 14, each group) following treatment with LCP, FCP, and clarithromycin on different days post-infection. Error bars represent the standard deviation (SD) from three independent values. ^∗^*P* < 0.05 indicate statistical significant differences (Student’s *t*-test) between infected control and treated groups. ^∗∗^*P* < 0.05 indicate statistical significant differences between FCP treated and other treated groups (i.e., LCP and clarithromycin treated).

Neutrophils are the first cells to reach the site of infection or inflammation. MPO is then produced by neutrophils into the extracellular space during degranulation. So as to assess the levels of inflammation or bacterial load MPO levels were measured, and results are presented in **Figure 6**. Highest MPO levels were present in untreated animals on all days with peak MPO activity of 2.81 units/ml recorded on day 3, that decreased on subsequent days. In case of FCP treated mice, peak MPO activity of 2.41 units/ml was observed on day 1, followed by significant decline thereafter as compared to untreated mice with minimal value of less than 1 unit/ml measured on day 10. However, maximum reduction in MPO levels was seen in mice receiving LCP as it showed peak MPO levels of 1.77 units/ml on day 1 with minimum MPO activity of 0.211 units/ml recorded on day 7 which is significantly less (*P* < 0.05) than activity present in untreated and FCP treated mice Lower levels of MPO activity observed on subsequent days in LCP treated group correlated well with lesser bacterial load seen after treatment. Clarithromycin treated mice also showed significant reduction (*P* < 0.05) in MPO enzyme activity as compared to infection control group and FCP treated group. Thus, these results indicate that neutrophil migration was maximum in untreated animals, while neutrophil migration upon phage and antibiotic treatment decreased due to the decrease in bacterial infection at the site.

##### Masson’s trichrome staining

**FIGURE 7:**
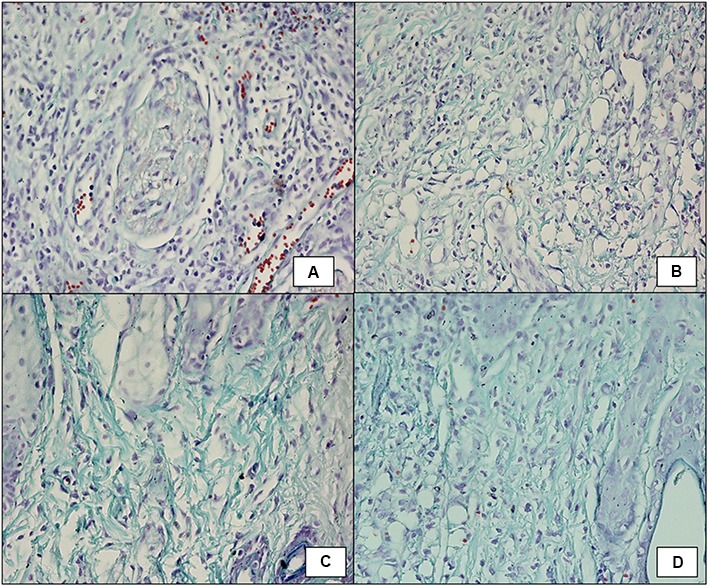
Masson’s Trichrome staining (100X) showing collagen formation on day 5 post-infection and treatment in wound tissue of **(A)** infected control mice (diabetic untreated mice) showed fibroblastic condensation along with infiltration of neutrophils. **(B)** Mice treated with FCP showed intermediate stage of collagen formation along with presence of mature collagen fibrils. **(C)** Mice treated with LCP showed thin fibrils of mature collagen present with decreased vascularity and reduced inflammatory cells. **(D)** Mice treated with clarithromycin showed negligible inflamation along with presence of mature collagen fibers, indicating significant wound healing.

Wound tissue was taken on day 5 post-infection from all the groups. The tissue of untreated diabetic mice showed little collagen formation as seen in **Figure 7A**. The faint green area seen in the figure depicts early stages of collagen formation. Mostly, growing fibroblast was present in between the majority of blood vessels, capillaries and inflammatory cells, but much less mature collagen fibers were visible with fibroblastic condensation, showing delayed and early stage of wound healing. FCP treated mice (**Figure 7B**) showed growing fibroblasts present between blood vessels and inflammatory cells and thin collagen fibrils, which is an intermediate stage of collagen formation, were common. Mature collagen was also present. The amount of collagen formed was comparatively greater than the untreated mice and less than the cocktail and antibiotic treated groups. LCP treated animals showed maximum formation of mature collagen among all the groups (**Figure 7C**). As is clearly evident that collagen recovered from individual thin fibrils in the dermis into thick bundles or fibers, indicating that mostly mature collagen was present. This correlated well with the less bacterial bioburden on day 5. Together with decreased vascularity and reduced inflammatory cells, the results suggest late stage of wound healing because collagen formation is one of the essential markers of wound healing. Clarithromycin treated animal group also showed presence of mature thick collagen fibers but thin collagen fibrils were comparatively more than LCP treated mice (**Figure 7D**).

##### Histopathological examination

**FIGURE 8:**
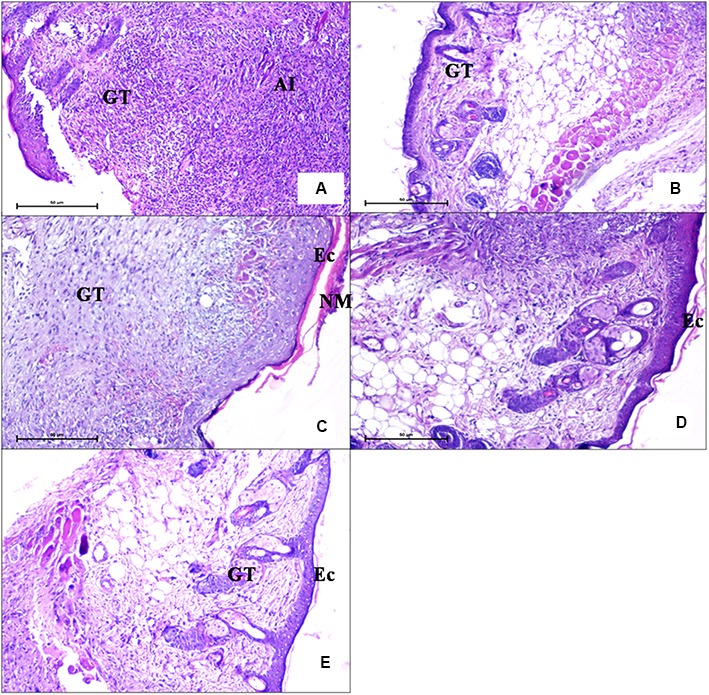
Photomicrographs of wound tissue of **(A)** an infection control mice (diabetic untreated mice) (H and E 100X) showed presence of acute inflammation. **(B)** Mice treated with FCP showed less infiltration of neutrophils and presence of granulation tissue **(C,D)** Mice treated with LCP showed epidermal hyperplasia coupled with reduced inflammation. **(E)** Mice treated with clarithromycin showed nearly normal epidermis with granulation tissue and sweat glands, indicating wound healing (GT, granulation tissue; AI, acute inflammatory; NM, necrotic material; EC, eschar).

For histopathological examination, wound tissue was taken on day 5 in order to study tissue damage and extent of wound healing in all the groups. **Figure 8A** depicts wound tissue of untreated animals (group 1). Acute inflammation associated with surface ulceration and breached epidermal layer was seen. A zone of acute inflammatory (AI) reaction with large numbers of infiltrating inflammatory cells and development of vascular granulation tissue (GT) showing large number of capillaries, blood vessels, and inflammatory cells is also visible. But mice receiving FCP (group 2) showed formation of epidermal buds extending toward dermis (**Figure 8B**), growing fibroblasts in the granulation tissue and collagen formation, indicating healing along with presence of less inflammatory cells. Hair follicles were also visible indicating regeneration of damaged skin. Similarly, clarithromycin treated mice (group 4) showed progressively reduced inflammation with few neutrophils present in the wound tissue (**Figure 8E**). Presence of granulation tissue with fibroblasts, collagen formation, and epidermal buds indicated rapid and significant wound healing in clarithromycin treated group. However, maximum healing with minimum tissue infiltration and damage was clearly visible in the wound tissue of mice treated with LCP as compared to free phage cocktail treated groups (**Figures 8C,D**). In LCP treated group a thick eschar (Ec) over the surface consisting of necrosed cells in dense eosinophillic coagulated protein background was visible. Epidermal healing was accompanied with hyperplasia of epidermis with buds and streaks extending deep into the dermis. Fewer growing fibroblasts and mostly collagen was seen in the granulation tissue with decreased vascularity and reduced inflammatory cells. Presence of hair follicles was also evident. These results indicate significant wound healing by day 5. This effect might be due to the higher number of phages present at the wound site as compared to phage titre in case of FCP treated animals.

## Discussion

Bacteriophage therapy represents an attractive treatment option for the eradication of *S. aureus* induced diabetic wound infections. Sunagar et al. (2010) reported significant protection in diabetic mice (90% survival rate) from lethal bacteremia following single injection of *S. aureus* specific phage (phage GRCS). Similarly, VinodKumar et al. (2011) demonstrated that wounds of diabetic rats which were infected with *S. aureus* were sterilized within 8 days after receiving monophage treatment. Chhibber et al. (2013) focused on the synergistic use of phage and antibiotic in the effective resolution of hindpaw infection in diabetic mice. However, in all of these studies monophage therapy was employed. Monophage based therapies suffer from the drawback of easy emergence of phage resistant bacterial mutants as well as their inability to cover a broad range of possible host targets (Scott et al., 2007; Labrie et al., 2010; Chadha et al., 2016). Thus, use of phage cocktail by incorporating different phages covering the broad host range can provide an effective solution that can be readily delievered without any delay, making it potentialy more effective clinically than monophage therapy (Chan et al., 2013).

In the present study, two already characterized *S. aureus* specific lytic phages, i.e., MR-5 and MR-10 (Kaur et al., 2012; Chhibber et al., 2013) belonging to *Myoviridae* family, were used to study therapeutic efficacy in resolving diabetic wound infection. The main focus of this study was to overcome two major drawbacks of phage therapy. Firstly, therapeutic efficacy of phage cocktail therapy (FCP) was studied and compared with monophage therapy in treatment of MRSA mediated wound infection. The results indicated that when phages were used singly or in cocktail (FCP) there was 100% protection as compared to untreated controls. However, more rapid reduction in bioburden and faster wound healing with FCP treated animals.

Secondly, the problem of potentially low phage retention and persistence *in vivo* was addressed by the use of lipid based carriers, i.e., liposomes. On studying the phage titer post-infection, it was found that phage titer dropped to 10^5^ PFU/ml from an initial titer of 10^9^ PFU/50 μl within 24 h of phage administration, resulting in presence of low phage number around the wound site. This low titer could be due to phage inactivation by skin’s extensive armamentarium of immune-competent cells, that contributed toward delayed phage effect (Merril et al., 1996; Keen, 2012; Abedon et al., 2014). The wound bio-burden initially increased as only 10^5^ phages were present at the wound site on day 1. The lower number of phage particles probably was unable to tackle the high bacterial load of actively multiplying bacterial population. But as phage growth occurred at the expense of host bacteria, this prevented further increases in bacterial count. This delayed protective response needs to be addressed by preventing rapid phage inactivation and clearance from infection site. Although past workers have made attempts to overcome this problem, however, each approach has its own limitations.

### Liposome Entrapment

Our laboratory has for the first time reported successful entrapment of phages within suitable lipid based delivery system, i.e., liposomes that are biocompatible and help to maintain phage titer within *in vivo* system (Singla et al., 2015; Chadha et al., 2017). In another study, encapsulation of *Salmonella* specific phages led to increased stability, prolonged intestinal residence and enhanced therapeutic efficacy (Colom et al., 2015). Liposomes have been regarded as effective DDS due to their GRAS (generally regarded as safe) status, high biocompatibility and high diffusivity in skin as compared to bare drugs (Verma et al., 2003). For drug delivery application liposomes are usually unilamellar and range in diameter from 100 to 300 nm. Large liposomes are rapidly removed from the blood circulation (Barenholz, 1998). Therefore, it is very important to optimize the liposome formulation in terms of size so as to maximize drug loading and minimize leakage and rapid clearance from body. Finally, the cocktail of phages MR-5 and MR-10 (10^9^ PFU/50 μl) (1:1) was entrapped within the liposome after optimizing the conditions required for their production and stability. Cocktail entrapped liposomal formulation (LCP) was of unilamellar nature, devoid of any observed aggregation, having a size of 212 nm and exhibited high entrapment efficiency of 87%. Stability studies in terms of size were conducted over a 9 week long period at different temperatures. The liposomal formulation was found to be most stable at 4°C without any physical changes or reduction in number of entrapped phages during storage period. However, at 37°C, an increase in particle size and aggregation was seen in the suspension. Leakage of entrapped drug from the liposomal formulation during storage before administration can lead to poor action of the administered drug (Nallamothu et al., 2006). The results are in consonance with the previous findings of Parmar et al. (2010) who showed that high temperature caused increase in size and aggregation as well as leakage of drug from liposomal formulation of budesonide.

The therapeutic efficacy of LCP was evaluated in Study 2. The wound bio-burden correlated well with the visible healing of the wound. No mortality occurred in any of the treatment groups. Maximum reduction was obtained on day 3 in mice treated with liposome entrapped cocktail as compared to infection control group. The entire process of wound healing and re-epithelization was faster and quicker in mice receiving LCP as compared to both untreated diabetic mice as well as animals that received FCP. No phage was present after day 10 in the wound tissue. However, phage titer was higher on all subsequent days in mice treated with liposome entrapped phage cocktail. A significant enhancement of 2 log was observed in LCP mice, clearly indicating that drop in initial titer and phage viability could be prevented by phage entrapment within liposomes. Also, as noted, workers in the past have shown that use of DDS improves the pharmacokinetics and bio-distribution of the associated drug (Allen and Cullis, 2004; Zhang et al., 2013). In addition, efficient uptake of encapsulated bacteriophage by eukaryotic cells has also been reported (Nieth et al., 2015; Singla et al., 2016).

### Faster Wound Healing

Wound contraction also demonstrated comparatively rapid healing (measured in terms of wound size) in all the treated groups as compared to infection control mice. Less number of days required for healing and obtaining sterile wounds was due to increased phage titer at wound site in LCP treated mice. Liposomes provided a depot effect and prevented phages from getting cleared off rapidly from the site. This rapid wound healing by day 9 also correlated well with the maximum deposition of mature collagen fiber in wound tissue by day 5 in animals treated with LCP. Mature collagen is an essential marker of wound healing and represents later stages of wound healing (Braiman-Wilksman et al., 2007; Suvik and Effendy, 2012). Histopathological examination of wound tissue of animals also supported these findings, and confirmed that those mice that received LCP showed minimal tissue damage and inflammation as compared to other groups. Epidermal healing and good amount of granulation tissue indicated significant wound healing by day 5. Besides this, maximum decrease in tissue MPO levels was also seen in group receiving LCP. This could be due to the broad spectrum effect of phage cocktail against MRSA, which efficiently arrested growth of invading bacteria at the site of injury, leading to decrease in neutrophil accumulation that correlated well with tissue healing.

From these observations it is concluded that although FCPs is a better option than monophage therapy, rapid inactivation of phages subsequently affects their ability in clearing the infectious agent. Liposomes are suitable delivery systems that help to increase the therapeutic efficacy of phages by improving their stability and delayed clearance by allowing timely release of phages at the infection site, leading to faster healing. The findings of this study thus provide new insights in the treatment of diabetic wound infection not explored by past workers. The use of liposome entrapped phage preparation is an attractive option.

## Author Contributions

SC: conceived and designed the experiments/contributed reagents/materials/analysis tools. JK and SK: performed the experiments. SC and SK: analyzed the data. SC, JK, and SK: wrote the paper.

## Conflict of Interest Statement

The authors declare that the research was conducted in the absence of any commercial or financial relationships that could be construed as a potential conflict of interest.

## Footnotes

1 http://www.ihcworld.com/_protocols/special_stains/masson_trichrome.htm
